# Distribution of coronary artery disease in acute coronary syndrome patients with diabetes mellitus

**DOI:** 10.1186/1749-8090-8-S1-P151

**Published:** 2013-09-11

**Authors:** D Gulin, E Galić, L Vrbanić, K Kordić, J Šikić Vagić

**Affiliations:** 1 Depatrment of Cardiovascular Disease, University Hospital Sveti Duh, Zagreb, Croatia; 2 University J.J. Strossmayera, Osijek, Croatia

## Background

Diabetes mellitus (DM) type II is an important risk factor for coronary heart disease (CAD), regardless of age and other comorbidities. In this study we investigate the distribution of CAD in patients with acute coronary syndrome (ACS) and diabetes who underwent primary percutaneous coronary intervention (pPCI).

## Methods

The study included 125 patients who were admitted to Department of Cardiovascular Diseases, University Hospital “Sveti Duh” for ACS in 2012 and treated with PCI. Analysis of coronarograms was conducted to determine the prevalence and distribution of CAD in patients with DM.

## Results

Leading diagnosis at admission in ACS patients with DM and without DM was STEMI (53%, 55% respectfully). According to the ACC/AHA classification of coronary lesions patients with DM had 56% of type B lesion, 41% of type C lesion and 3% of type A lesion. In our study, the largest number of significant stenosis was observed in the proximal and middle segment of LAD: 74% of patients with DM and 60% in patients without DM. In patients with DM single-vessel CAD was observed in 26%, two-vessel in 41% and three-vessel in 32%, whereas in patients without DM, 52% single-vessel CAD, 30% two-vessel and 18 % three-vessel CAD.

## Conclusion

Most of the studies showed that patients with DM are more likely to have diffuse distribution of CAD. Results of studies about the association of DM with the location of the lesion in CAD is also contradictory, in some studies was observed a higher incidence of proximal, and in other distal lesions. In our study, mostly affected was LAD, usually its proximal and middle segment. Also, RCA and ACx were more affected in the proximal and middle segment. The study found a higher prevalence of type C lesions and a higher prevalence of three-vessel CAD in patients with DM which confirms previous findings that patients with DM usually have diffuse CAD.

